# A multivariable probability score integrating routine immunoassay results for the immunological assessment of antisynthetase syndrome

**DOI:** 10.3389/fimmu.2026.1832694

**Published:** 2026-05-29

**Authors:** Juan Francisco Luchoro, Maria Torradeflot, Carmen Domènech, Lucía Marcelo-Torreras, Mario Framil, Raül Jordà-Sánchez, Andrea García-López, Sergio Prieto-González, Estíbaliz Ruiz-Ortiz

**Affiliations:** 1 Immunology Department, Centre de Diagnòstic Biomèdic (CDB), Hospital Clínic de Barcelona, Barcelona, Catalonia, Spain; 2 Department of Autoimmune Diseases, Reference Centre for Systemic Autoimmune Diseases (UEC/CSUR) of the Catalan and Spanish Health Systems-Member of ERNReCONNET, Hospital Clínic de Barcelona, Institut d'Investigacions Biomèdiques August Pi i Sunyer (IDIBAPS), Universitat de Barcelona, Catalonia, Spain

**Keywords:** Anti-aminoacyl–tRNA synthetase autoantibodies, anti-Ro52 autoantibodies, antisynthetase syndrome, autoantibody interpretation, HEp-2 indirect immunofluorescence, line-blot immunoassay, multivariable logistic regression, probability-based diagnostic score

## Abstract

**Background:**

Accurate detection of anti-aminoacyl–tRNA synthetase autoantibodies (ASA) is central to the diagnosis of antisynthetase syndrome (ASyS). Although immunoprecipitation (IP) remains the historical reference method, its limited availability in routine clinical laboratories necessitates reliance on commercially available immunoassays. The diagnostic performance of these assays varies by specificity and cut-off interpretation. We aimed to improve the post-analytical interpretation of ASA positivity in routine practice.

**Methods:**

We conducted a retrospective single-center study including 125 patients with at least one positive ASA detected by line-blot immunoassay (LIA) between January 2023 and September 2025. Thirty-three patients fulfilled clinician-based diagnostic criteria for ASyS, while 92 served as controls. Additional immunological data included indirect immunofluorescence on HEp-2 cells (IIF-HEp-2) and anti-Ro52 autoantibodies. Variables associated with ASyS were incorporated into multivariable logistic regression models. A diagnostic probability score was derived from the final model and evaluated using receiver operating characteristic (ROC) curve analysis.

**Results:**

Semi-quantitative LIA intensity alone showed limited discriminatory capacity, particularly in cases with low-intensity bands. In contrast, compatible cytoplasmic IIF-HEp-2 patterns and anti-Ro52 co-positivity significantly improved discrimination. The final multivariable model integrating semi-quantitative LIA intensity, IIF-HEp-2 cytoplasmic pattern (AC-19/20), and anti-Ro52 co-positivity achieved an AUC of 0.94 (95% CI 0.89–0.99). Stratification into low-, intermediate-, and high-probability zones provided clinically interpretable diagnostic categories.

**Conclusion:**

In ASA-positive patients, probability-based integration of routinely available immunoassay results improves the interpretation of LIA positivity and enhances discrimination between ASyS and alternative diagnoses.

## 1 Introduction

Antisynthetase syndrome (ASyS) (1) is a rare autoimmune disorder within the spectrum of idiopathic inflammatory myopathies (IIM), characterized by a heterogeneous multisystem involvement. Clinically, it most commonly presents with muscle weakness, interstitial lung disease (ILD), arthritis, Raynaud’s phenomenon, mechanic’s hands and fever (2). Beyond its clinical manifestations, ASyS is defined by the presence of autoantibodies directed against aminoacyl-tRNA synthetases (ARSs), which constitute its central immunological hallmark.

ARSs are a family of enzymes essential for protein synthesis, catalyzing the attachment of specific amino acids to their cognate transfer RNAs (3). The identification of autoantibodies targeting these enzymes established the first link between ARSs and human autoimmune disease and led to the recognition of ASyS as a distinct clinical entity. The earliest anti-ARS autoantibody (ASA) was described in 1980 in patients with IIM (4), when the Jo-1 antigen was identified as histidyl-tRNA synthetase. Subsequently, autoantibodies against seven additional ARSs—ThrRS, AlaRS, GlyRS, IleRS, AsnRS, PheRS and TyrRS—were reported and designated anti-PL-7, anti-PL-12, anti-EJ, anti-OJ, anti-KS, anti-Zo and anti-HA, respectively (5–10). Additionally, autoantibodies targeting other ARS, such as CysRS, have recently been described using novel techniques (11). Together, these findings highlight the pivotal role of ASA in the diagnosis and classification of ASyS (12).

Immunoprecipitation (IP) remains the gold-standard method for detecting ASA. However, IP is labor-intensive, technically demanding, and therefore not routinely available in clinical laboratories. To overcome these limitations, commercially available antigen-specific assays—including enzyme-linked immunosorbent assays (ELISA) and line-blot immunoassays (LIA)—have gained widespread use. These methods provide a faster, more accessible, and more cost-effective alternative for detecting ASA (13). LIA allows the simultaneous assessment of multiple autoantibodies within a single test, facilitating broader serological profiling. Nevertheless, their diagnostic performance varies considerably by autoantibody specificity. False-positive results are frequently associated with biologically implausible findings, such as incongruent anti-cytoplasmic pattern by indirect immunofluorescence on HEp-2 cells (IIF-HEp-2), specifically patterns different to those associated with ASA (AC-19 or AC-20), or the detection of multiple specificities, which complicate clinical interpretation (13–15). Altogether, variability in sensitivity and specificity has been reported depending on the antigen and the assay used (13, 16).

In this context, additional serological markers that may refine diagnostic accuracy and clinical stratification are of particular interest. Anti-Ro52 (TRIM21) autoantibodies are frequently detected in patients with ASyS in association with ASA (17). Although anti-Ro52 is not classified as a myositis-specific autoantibody (MSA), its presence in ASyS has been consistently associated with clinically relevant phenotypes, especially more frequent and severe ILD and an increased risk of progressive disease (18). Importantly, anti-Ro52 autoantibodies are not mutually exclusive with ASA and therefore provide complementary rather than redundant information. Their inclusion in routine serological testing may enhance patient stratification and risk assessment in ASyS (17).

Taken together, these considerations underscore the need for diagnostic strategies that can reliably identify ASA using routinely available laboratory techniques, particularly when IP is not accessible. Therefore, the aim of this study was to develop and evaluate a laboratory-based probabilistic scoring system for the post-analytical interpretation of positive ASA results by LIA, using routinely available serological assays, to improve discrimination between clinically confirmed ASyS and alternative diagnoses in ASA-positive patients.

## 2 Methods

### 2.1 Study population

**Figure 1:**
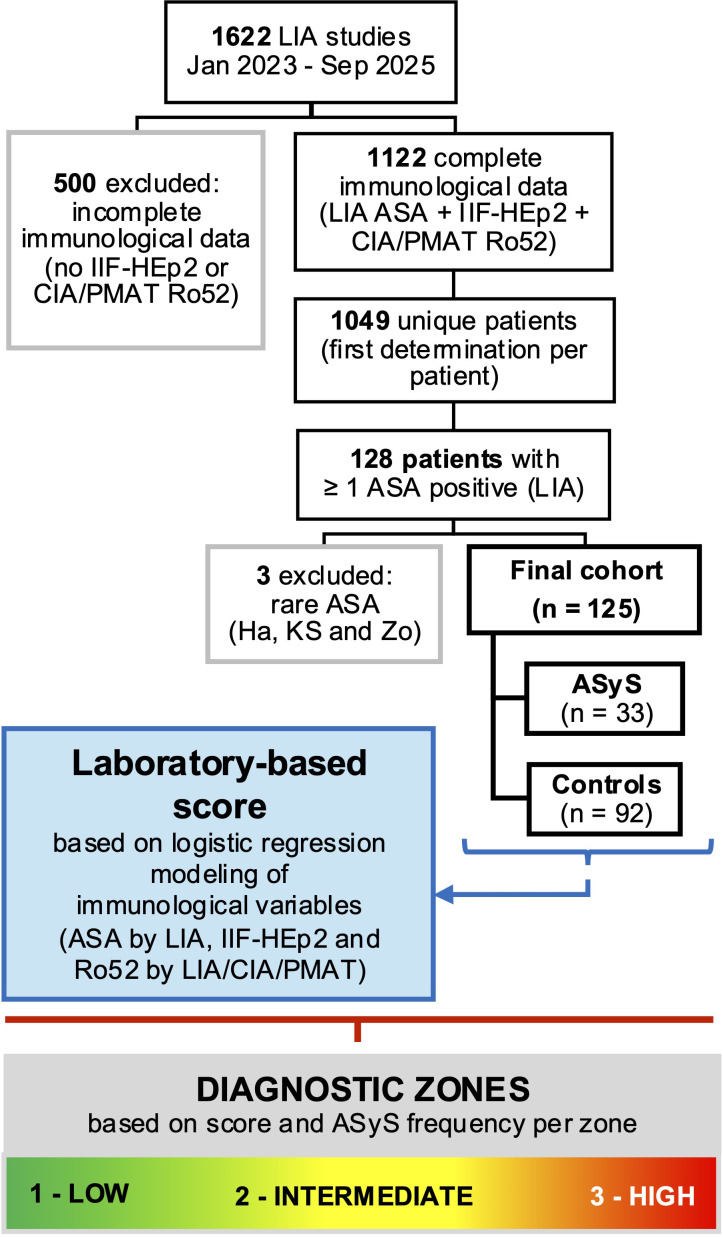
Workflow of patient classification, immunological testing, and development of a laboratory-based immunological scoring system for antisynthetase syndrome (ASyS). A total of 125 patients were classified according to final clinical diagnosis, underwent multimodal antibody testing, and were stratified into diagnostic probability zones based on an integrated immunological score. ASA, anti-synthetase antibodies, CIA, chemiluminescence immunoassay, IIF-HEp2, indirect immunofluorescence in HEp2 cells, LIA, line-blot immunoassay, PMAT, particle multi-analyte technology, Ro52, Anti-Ro52 antibodies.

This retrospective study was conducted using a laboratory database from Hospital Clínic de Barcelona and included all myositis LIA studies performed between January 2023 and September 2025. This database comprised patients evaluated for suspected IIM in whom an extended myositis autoantibody profile had been performed as part of routine clinical practice. A total of 1,622 LIA determinations were initially retrieved. Among these, 1,122 determinations additionally included IIF-HEp2 and anti-Ro52 results. After exclusion of repeated measurements, 1,049 unique patients were identified. To minimize potential bias related to longitudinal sampling, only the first available determination obtained during the diagnostic work-up of each patient was included in the analysis (**Figure 1**).

For the present analysis, patient selection was based exclusively on laboratory criteria rather than on clinical suspicion. Specifically, all patients with at least one positive ASA result detected by LIA were eligible for inclusion, regardless of signal intensity. This approach yielded 128 patients. Three patients were subsequently excluded due to isolated reactivity for rare antigens (KS, Ha, and Zo) included in the extended panel, which were insufficiently represented for meaningful analysis. The final analytical cohort therefore consisted of 125 patients. Accordingly, the model was developed and evaluated exclusively within an ASA-positive population and was not intended to estimate the diagnostic performance of ASA testing in unselected patients.

Patients were subsequently classified according to their final diagnosis, which was established independently according to multidisciplinary evaluation following the criteria proposed by the 273rd ENMC International Workshop for ASyS (19). Based on this assessment, 33 patients were diagnosed with ASyS, while the remaining patients (n=92) —despite ASA positivity—were classified as controls due to alternative diagnoses (**Supplementary Table 1**).

A schematic overview of the study population selection process is shown in **Figure 1**.

### 2.2 Sample testing

All immunological analyses were performed and validated in the Autoimmunity Unit of the Immunology Department at Hospital Clínic de Barcelona. The determination of ASA was performed using commercially available assays, including LIA (Euroimmun^®^), IIF-HEp-2 (Werfen^®^), and chemiluminescence immunoassay (CIA; QuantaFlash^®^, Werfen^®^) or particle-based multi-analyte technology (PMAT; Werfen^®^). CIA and PMAT were used for the detection of anti-Jo-1 and anti-Ro52 autoantibodies, according to test availability, depending on the testing period.

All assays were interpreted according to manufacturer-defined cut-offs, which were consistently applied throughout the study. Although these tests assess related immunological targets, they were considered separate analytical variables, as they are generated, processed, and reported independently, and are based on different analytical principles and platforms.

LIA testing was performed on the EUROBlotOne automated platform following the manufacturer’s instructions. Two different panels were used according to clinical request and availability: Autoimmune Inflammatory Myopathies 16 Ag et cN-1A IgG and Autoimmune Inflammatory Myopathies 20 Ag IgG. Each strip contains multiple recombinant antigens immobilized in discrete lines. The ARS included in each profile were Jo-1, PL-7, PL-12, EJ and OJ in the 16-antigen panel, and Jo-1, PL-7, PL-12, EJ, OJ, KS, Ha and Zo in the 20-antigen panel. Both profiles also include Ro52. For the purpose of this study, only ARS specificities with sufficient representation in the cohort were included in the analysis. Therefore, cases with isolated positivity for rare antigens exclusively present in the 20-antigen panel (KS, Ha and Zo) were excluded, and these specificities were not considered in the model.

Band intensity (BI) for each antigen was quantified using EUROLineScan software provided by the manufacturer. Each strip includes a positive control band (PCB); test validity required a PCB BI >50. Once validity was confirmed, antigen-specific BI values were interpreted according to the manufacturer’s semi-quantitative categories: 0–15 negative, 16–35 weakly positive (+), 36–70 moderately positive (++), and ≥71 strongly positive (+++). In cases of multiple ASA positivities, only the autoantibody with the highest intensity was retained for analysis, to prioritize the most clinically relevant signal and reduce the impact of low-intensity non-specific reactivity.

IIF-HEp-2 was performed using standard laboratory protocols. Slides were independently reviewed by a trained laboratory technician and a laboratory physician. A titer ≥1:80 was considered positive. Fluorescence patterns were classified according to the International Consensus on ANA Patterns (ICAP) (20). The ICAP classification includes both nuclear and cytoplasmic patterns; however, only cytoplasmic patterns—namely AC-19 and AC-20—are associated with the presence of ASA. For this reason, AC-19 and AC-20 cytoplasmic patterns were specifically recorded. For analytical purposes, results were categorized as positive with a compatible (AC-19/AC-20) pattern or as negative or positive with a non-compatible pattern.

### 2.3 Statistical analysis

Categorical variables were summarized as absolute numbers and percentages. Comparisons between patients with and without ASyS were performed using Fisher’s exact test, given the limited sample size and the presence of low expected cell counts in several comparisons.

Variables showing a statistically significant association with ASyS in bivariate analysis were entered into multivariable logistic regression models, with the final clinical diagnosis of ASyS as the dependent variable, to identify laboratory markers independently associated with the outcome. When several competing models were constructed, model comparison was performed using analysis of variance (ANOVA) based on likelihood ratio tests, and the model with the best balance between goodness of fit and parsimony was selected. Results were expressed as β-coefficients with 95% confidence intervals (95% CI). A two-sided p value <0.05 was considered statistically significant.

The diagnostic performance of the selected model was evaluated using receiver operating characteristic (ROC) curve analysis. The optimal cut-off value was determined by maximizing the Youden index. Discriminative ability was summarized by the area under the ROC curve (AUC) with corresponding 95% confidence intervals. Given the study design, which was restricted to ASA-positive patients, classical diagnostic performance metrics –such as sensitivity, specificity, positive predictive value, and negative predictive value in an unselected population– were not estimated.

All statistical analyses were performed using R software (version 4.5.1; R Foundation for Statistical Computing, Vienna, Austria). Logistic regression modeling and ROC curve analyses were conducted using established R packages.

### 2.4 Derivation of the laboratory-based diagnostic score

A laboratory-based diagnostic score was derived directly from a multivariable logistic regression, including only immunological variables. The model incorporated LIA intensity (ordinal variable: +, ++, +++), cytoplasmic ANA patterns (AC-19/AC-20), and anti-Ro52 co-positivity as binary variables. For each patient, the predicted probability of ASyS was calculated using the regression equation and expressed as a percentage (**Figure 1**). This probability should be interpreted as the likelihood of clinically confirmed ASyS conditional on ASA positivity by LIA, rather than as an absolute disease probability in the general suspected-IIM population.

## 3 Results

### 3.1 General characteristics

A total of 125 patients were included in the analysis. Among them, 33 patients (26.4%) fulfilled the diagnostic criteria for ASyS. The remaining 92 patients (73.6%), despite presenting ASA positivity, were diagnosed with alternative conditions (**Supplementary Table 1**) and were therefore classified as controls. Demographic characteristics and key immunological features stratified by diagnostic groups are summarized in **Table 1**.

**Table 1:** Demographic characteristics and key immunological features stratified by diagnostic groups.

Characteristic	N	Controls N =92	ASyS patients N = 33	P-value^1^
Age	125	69 (56–76)	67 (50–74)	0.3
Gender	125			
Female	63	40 (43%)	23 (70%)	**0.017**
Male	62	52 (57%)	10 (30%)	
ASA by LIA	125			
+	59	57 (62%)	2 (6.1%)	**<0.001**
++	30	26 (28%)	4 (12%)	
+++	36	9 (9.8%)	27 (82%)	
ANA detection – Cytoplasmic patterns (IIF−HEp−2)	125			
Negative or non-compatible	86	79 (86%)	7 (21%)	**<0.001**
Compatible (AC-19/20)	37	13 (14%)	26 (79%)	
Anti-Ro52 co-positivity (by LIA/CIA/PMAT)	125	13 (14%)	24 (73%)	**<0.001**

Values are expressed as median (Q1–Q3) or n (%). ASyS, anti-synthetase syndrome; CIA, chemiluminescence immunoassay; IIF-HEp-2, indirect immunofluorescence on HEp-2 cells; LIA, line immunoassay; PMAT, particle-based multi-analyte technology. ^1^Wilcoxon rank-sum test for continuous variables; Pearson’s chi-squared test for categorical variables. Statistically significant p-values are shown in bold.

No significant differences in age distribution were observed between groups. A higher proportion of female patients was identified in the ASyS group (p=0.017). ASA reactivity intensity assessed by LIA was significantly associated with ASyS diagnosis (p<0.001). Among non-ASyS group, low-intensity results (+ or ++) were more frequently observed than high-intensity (+++) results.

**Figure 2:**
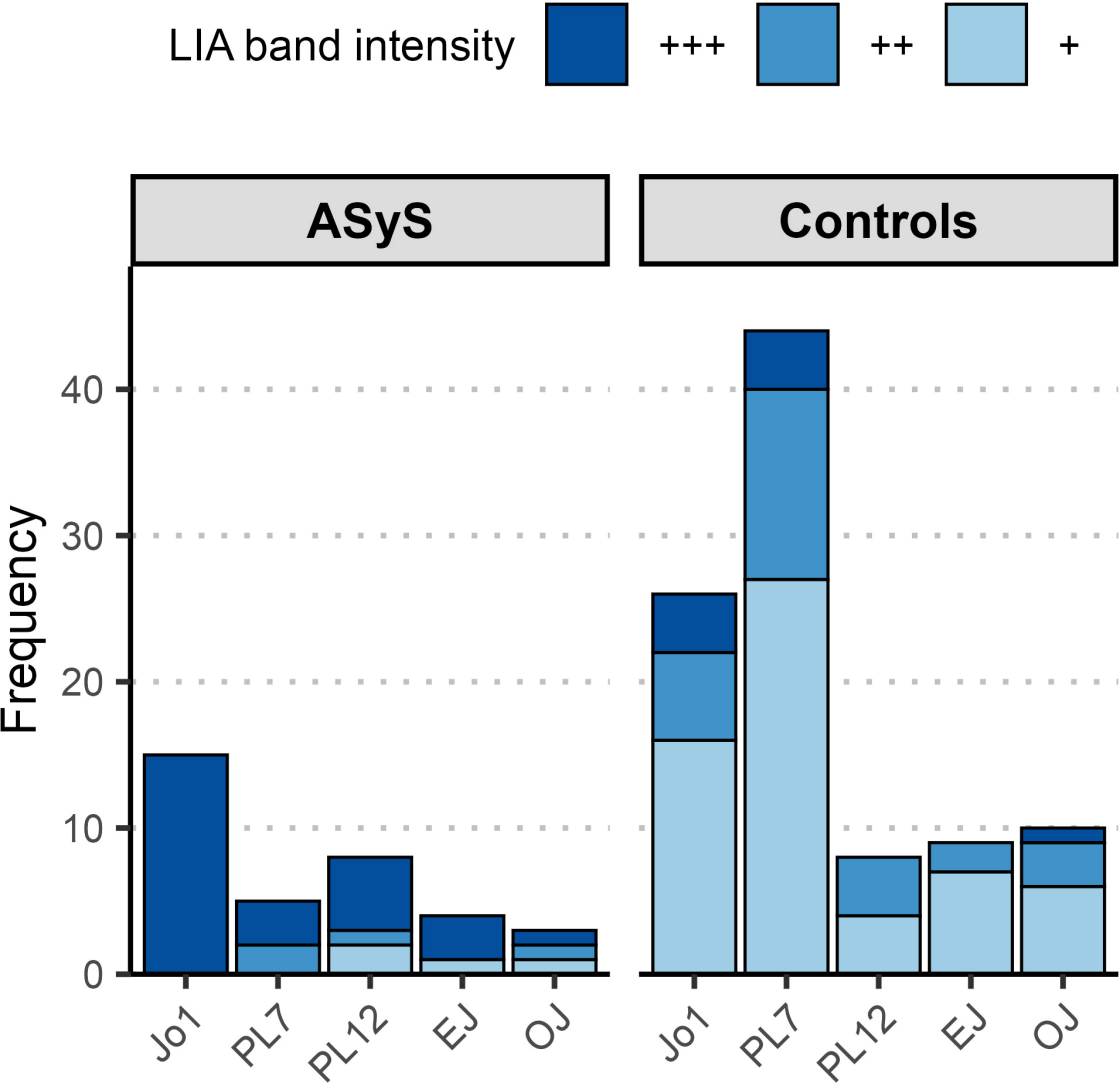
Distribution of antisynthetase autoantibody (ASA) specificities and corresponding line-blot assay band intensities stratified by diagnostic group. Stacked bar plots showing the distribution of weak (+), moderate (++), and strong (+++) band intensities for each ASA specificity (Jo-1, PL-7, PL-12, EJ, OJ) in antisynthetase syndrome (ASyS) patients and controls. Bar heights represent the absolute frequency of each autoantibody, while the stacked segments indicate the contribution of each intensity category. A higher proportion of strong-positive (+++) bands is observed in ASyS patients, whereas controls are predominantly characterized by weak- and moderate-intensity reactivities.

The distribution of ASA specificities and their corresponding LIA intensity levels is shown in **Figure 2**, **Table 2**. A total of 132 positive ASA reactivities were identified across the cohort, reflecting the presence of multiple positivities in some patients. Although all detected ASA specificities were initially recorded, for analytical purposes a single reactivity per patient was selected based on the highest LIA intensity. Differences in the distribution of intensity levels were observed across autoantibody specificities. Anti-Jo-1 showed a higher proportion of strong-positive (+++) reactivity (46.3%), whereas PL-7 -the most frequently detected autoantibody (37.1%) of all positivities- was predominantly associated with weak-positive (+) results (55.1%).

**Table 2:** Distribution of positive specificities detected by LIA.

Result	EJ	Jo1	OJ	PL12	PL7	Total
+	8 (61.5%)	16 (39.0%)	7 (53.8%)	6 (37.5%)	27 (55.1%)	64
++	2 (15.4%)	6 (14.6%)	4 (30.8%)	5 (31.2%)	15 (30.6%)	32
+++	3 (23.1%)	19 (46.3%)	2 (15.4%)	5 (31.2%)	7 (14.3%)	36
Total	13	41	13	16	49	132

Values are expressed as number of positive samples for each specificity, with percentages calculated relative to the total number of positive results for that specificity. Band intensity categories were defined according to manufacturer criteria as weak positive (+), moderately positive (++), and strongly positive (+++).

Regarding IIF-HEp-2 results, most non-ASyS patients with a positive result by LIA showed an incompatible pattern (79/92, 85.8%, p<0.001), whereas the majority of ASyS patients presented a compatible cytoplasmic pattern (AC-19/AC-20) (26/33, 78.8%, p<0.001). Similarly, anti-Ro52 co-positivity was significantly more frequent in ASyS patients than in controls (72.7% vs 14.1%, p<0.001).

When stratified according to LIA intensity (**Table 3**), the distribution of ANA cytoplasmic patterns and anti-Ro52 co-positivity differed across intensity categories. In ASyS patients, the proportion of compatible ANA patterns increased with higher LIA intensity, reaching 85.2% in strong-positive (+++) results. In contrast, compatible cytoplasmic patterns (AC-19/AC-20) remained infrequent across all intensity levels in controls. A similar trend was observed for anti-Ro52 co-positivity, which was more frequent in ASyS patients, particularly at higher LIA intensity levels (77.8%, p<0.001), while remaining low in controls across all categories (**Table 3**).

**Table 3:** Distribution of ANA cytoplasmic pattern compatibility and anti-Ro52 co-positivity according to LIA intensity and diagnostic group.

Group	LIA	N	ANA compatible (AC-19/20)	p-value (ANA)	Anti-Ro52 co-positivity	P-value (anti-Ro52)
ASyS	+	2	0 (0%)	–	1 (50%)	–
Controls	+	57	8 (14%)		8 (14%)	
ASyS	++	4	3 (75%)	0.034	2 (50%)	0.169
Controls	++	26	4 (15.4%)		4 (15.4%)	
ASyS	+++	27	23 (85.2%)	<0.001	21 (77.8%)	<0.001
Controls	+++	9	1 (11.1%)		1 (11.1%)	

Data are presented as n (%). Percentages are calculated within each LIA intensity category and diagnostic group. Comparisons between ASyS and control patients within each intensity level were performed using Fisher’s exact test; p-values are shown where applicable.

### 3.2 Logistic regression models - establishing a laboratory-based immunologic diagnostic predictor

Multiple logistic regression models were constructed for the main diagnostic assays used. A univariable model including only LIA was first developed, followed by two multivariable models integrating LIA with IIF-HEp-2, and LIA with both IIF-HEp-2 and anti-Ro52 co-positivity, with the aim of improving overall discriminative performance.

The univariable model showed a moderate ability to predict disease status, whereas the multivariable models demonstrated improved discrimination between ASyS and control patients. Model comparison using likelihood ratio tests (ANOVA) confirmed the superior performance of the combined models. The final model, including LIA intensity, cytoplasmic ANA pattern (AC-19/AC-20), and anti-Ro52 co-positivity, was selected based on the best balance between goodness of fit and parsimony. A summary of the regression coefficients is provided in **Table 4**.

**Table 4:** Multivariable logistic regression model for anti-synthetase syndrome diagnosis.

Characteristic	β coefficient	95% CI	P-value
ASA result by LIA			
+	—	—	
++	1.2	-0.52, 3.1	0.2
+++	3.5	2.0, 5.3	<0.001
ANA detection – cytoplasmic patterns (IIF−HEp−2)			
Negative or positive without AC-19/20 pattern	—	—	
Compatible (AC-19/20)	1.8	0.50, 3.2	0.007
Ro52 co-positivity when ASA is positive by LIA			
No	—	—	
Yes	1.6	0.26, 3.1	0.021

β coefficients are shown for each variable included in the final multivariable logistic regression model. Reference categories were: ASA-negative (for LIA), negative or non-compatible indirect immunofluorescence (IIF) HEp-2 pattern (for IIF), and absence of anti-Ro52 co-positivity (for anti-Ro52). ASA, anti–aminoacyl-tRNA synthetase autoantibodies; CI, confidence interval; HEp-2, human epithelial type 2 cells; LIA, line immunoassay.

**Figure 3:**
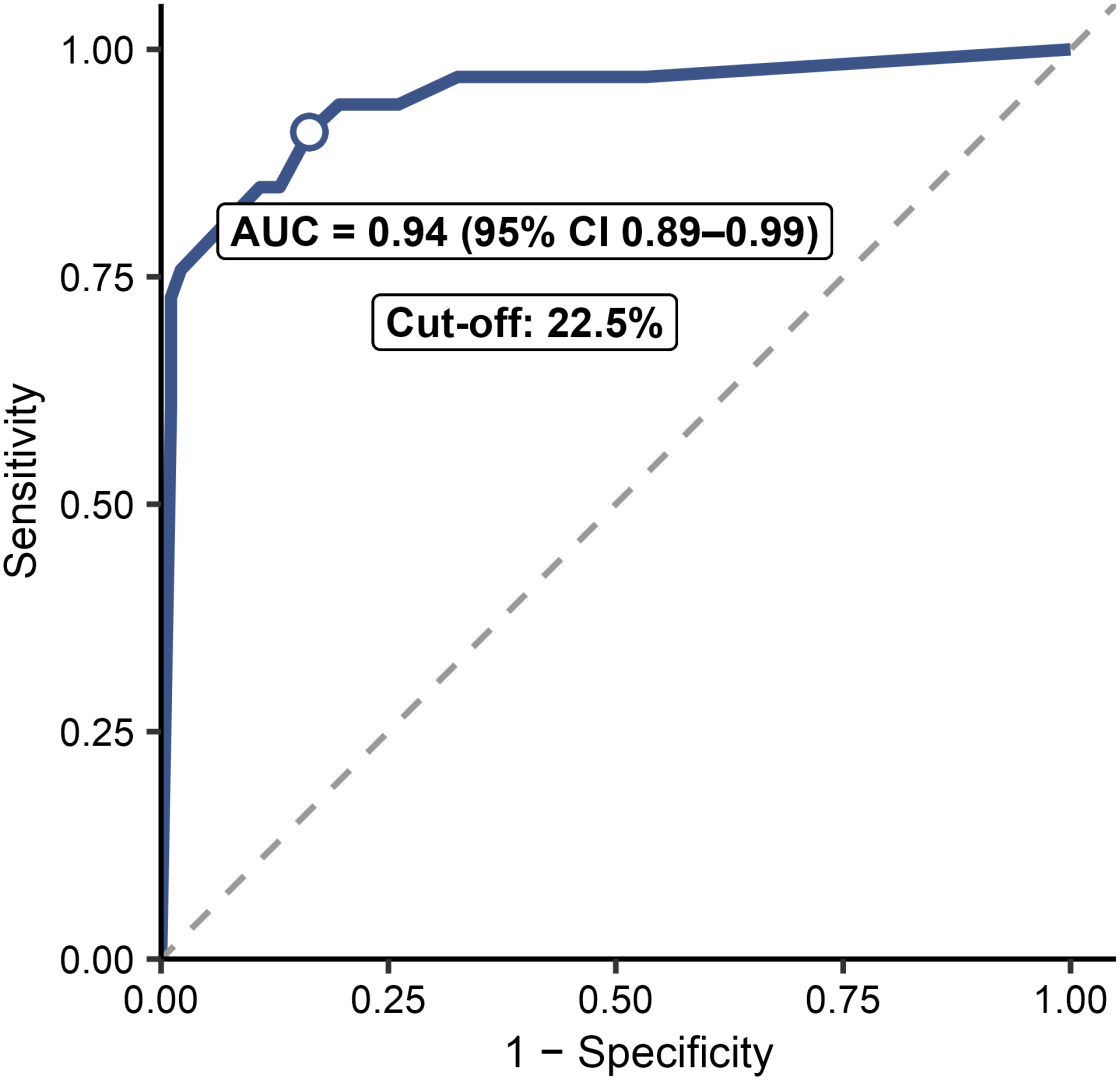
Receiver operating characteristic (ROC) curve of the final multivariable logistic regression model for the discrimination of antisynthetase syndrome within an ASA-positive cohort. The ROC curve illustrates the discriminative performance of the laboratory-based score integrating semi-quantitative LIA intensity, IIF-HEp-2 cytoplasmic pattern (AC-19/20), and anti-Ro52 co-positivity. The area under the curve (AUC) was 0.94 (95% CI 0.89–0.99). The optimal cut-off determined by Youden’s index was 22.5%.

The laboratory-based diagnostic score corresponds to the predicted probability of ASyS derived directly from the logistic regression equation, based on the weighted contribution (β-coefficients) of each variable. Probabilities were expressed as percentages to facilitate clinical interpretation. The next step was to evaluate the diagnostic performance of the individual assays and the derived model. To further assess the discriminatory capacity of the scoring system, a ROC analysis was performed, enabling the identification of the optimal cut-off point for predicting a confirmed ASyS diagnosis. Within the selected ASA-positive cohort, the AUC of the score was 0.947 (95% CI 0.895-0.999), with an optimal cut-off of 22.5% according to Youden’s index (**Figure 3**).

### 3.3 Diagnostic zones based on the probability of an immunological ASyS diagnosis

Given the heterogeneity of the study population and the variability in pre-test probabilities across clinical settings, a single universal cut-off was not adopted. Instead, predicted probabilities derived from the final logistic regression model were stratified into three diagnostic zones based on score distribution and ROC analysis.

The lower boundary was informed by the ROC-derived optimal cut-off (Youden index=22.5%), representing the point of maximal combined sensitivity and specificity. Based on this reference, broader probability intervals were defined to facilitate clinical interpretation and to avoid forced dichotomization. Specifically, thresholds of <25%, 25–70%, and >70% were selected to define low-, intermediate-, and high-probability categories, respectively.

The low-probability zone (<25%) included 85 patients, of whom 5 were diagnosed with ASyS (5/85, 5.9%). The intermediate-probability zone (25–70%) comprised 15 patients, with 4 confirmed ASyS cases (4/15, 26.7%). The high-probability zone (>70%) included 25 patients, 24 of whom fulfilled diagnostic criteria for ASyS (24/25, 96.0%) (**Table 5**).

**Table 5:** Diagnostic probability zone.

Diagnostic zone	n	ASyS confirmed	Positivity rate (%)	Mean score (%, SD)
1 - Low probability (<25%)	85	5	5.9	5.4% (6.4)
2 - Medium probability (25-70%)	15	4	26.7	37.7% (10.0)
3 - High probability (>70%)	25	24	96.0	91.4% (7.1)

Diagnostic zones were defined based on the distribution of predicted probabilities and ROC-derived thresholds. Positivity rate corresponds to the percentage of confirmed ASyS cases within each probability category.

## 4 Discussion

In this study, we developed a laboratory-based immunological scoring system aimed at improving the interpretation of ASA positivity by discriminating between results associated with a high likelihood of ASyS and those more likely to represent clinically non-relevant or false-positive findings. The proposed score is based exclusively on results obtained from routinely performed and commercially available laboratory assays. By integrating LIA intensity, IIF-HEp-2 cytoplasmic patterns, and anti-Ro52 co-positivity into a multivariable logistic regression model, the score demonstrated high discriminative performance within the studied cohort. These findings support the concept that a structured, probability-based interpretation of routinely available immunoassays can improve the distinction between ASyS and alternative diagnoses in real-world laboratory settings, particularly in the absence of IP.

IP has historically been considered the gold-standard method for ASA detection. However, its technical complexity, limited availability, and lack of inter-laboratory standardization restrict its use in routine clinical practice (21, 22). In this context, our results are not intended to replace IP, but rather to support an integrated interpretation of standardized immunoassays that can provide clinically useful diagnostic information when IP is not available. Moreover, even in settings where IP can be performed, this approach may help to prioritize its use for selected, diagnostically challenging cases in which the additional information provided by this technique is most likely to be clinically informative. Importantly, in a proportion of cases, the application of the proposed score may be sufficient to reasonably exclude a weakly positive ASA result as clinically relevant, thereby avoiding unnecessary IP testing.

Consistent with previous reports, individual immunological assays in our cohort demonstrated reasonable diagnostic performance but were insufficient as standalone tools. Regarding LIA, several studies report clinically relevant false-positive rates when manufacturer cut-offs are applied, particularly among weak-positive bands, which show markedly lower positive predictive value than moderate/strong results (13, 23–25). In addition, concordance with IP also varies substantially depending on autoantibody specificity, reinforcing that an isolated LIA positivity (in the absence of other concordant immunological findings) should be interpreted with caution, especially for rarer ASA (13–15, 26). More recent evaluations confirm inter-assay variability across platforms and highlight the need for contextual interpretation of multiplex immunoassays (22, 27).

In our ASA-positive cohort, LIA intensity alone showed limited discriminatory value, particularly at lower intensity levels, whereas IIF-HEp-2 positive (AC-19/20) patterns and anti-Ro52 co-positivity added relevant discriminatory value between ASyS and non-ASyS cases. Cytoplasmic ANA patterns compatible with ASA (AC-19 and AC-20) have been increasingly recognized as diagnostically informative (20), and current reviews emphasize the importance of integrating serological findings with appropriate laboratory interpretation strategies (12, 21). Anti-Ro52 autoantibodies, although not disease-specific, are frequently detected in ASyS cohorts and may provide relevant contextual information when present alongside ASA (17, 18). We acknowledge that these variables are biologically related and should not be interpreted as fully independent causal predictors. However, the purpose of the model was not causal inference, but the pragmatic integration of complementary laboratory signals generated by independent analytical platforms.

The superior performance of the combined model underscores the complementary nature of these immunological markers. Rather than contributing independent binary signals, their diagnostic value increases when interpreted coherently. This principle is aligned with Dean et al. (28) recent recommendations advocating likelihood-based interpretation of autoantibody testing rather than dichotomous reporting. By integrating semi-quantitative LIA intensity with cytoplasmic IIF-HEp2 patterns and anti-Ro52 status within a multivariable logistic framework, the model leverages partially informative signals that may be insufficient in isolation, thereby improving overall discrimination within ASA-positive patients.

ASyS definitions and criteria remain heterogeneous, with multiple competing approaches largely focused on classification rather than probabilistic diagnosis (29–31). By contrast, our work aims to provide a laboratory-based probabilistic tool that can be applied at the diagnostic interface, particularly in settings without IP. This strategy is consistent with multiparametric approaches successfully implemented in other systemic autoimmune diseases, where weighted probabilistic models have demonstrated superior diagnostic performance compared to single-marker strategies (32–34). So, our approach follows successful precedents in other autoimmune diseases.

Although ROC analysis identifies an “optimal” cut-off based on Youden’s index, we intentionally avoided defining a single universal threshold for clinical application, as ASyS is evaluated across heterogeneous clinical settings with variable pre-test probabilities. In line with current recommendations advocating likelihood-based reasoning (28) we stratified predicted probabilities into low-, intermediate-, and high-probability zones. This approach preserves information contained in a continuous probability score and offers a clinically interpretable output that mirrors real-world reasoning: low-probability results may support alternative diagnostic pathways; intermediate-probability results may justify closer follow-up or complementary testing; and high-probability results increase diagnostic confidence and may facilitate earlier decision-making within multidisciplinary evaluation.

Several limitations of this study should be acknowledged. First, the model was developed and evaluated in a single-center cohort restricted to patients with at least one positive ASA result by LIA, representing a deliberate enrichment strategy. Consequently, the proposed score is not intended for screening purposes, and classical diagnostic performance metrics such as sensitivity, specificity, or negative predictive value in unselected populations cannot be reliably estimated. This design may also lead to an overestimation of the apparent discriminative performance compared to real-world clinical settings. Second, no external validation was performed, and the model was derived and tested within the same cohort. Therefore, its performance may be optimistic, and validation in independent populations and across different assay platforms will be essential, particularly given the known inter-assay variability in myositis autoantibody testing (13, 27). Third, IP, the historical reference method for ASA detection, was only available in a limited number of patients, precluding direct comparison with a gold standard. In addition, the absence of IP limits the ability to detect rare or previously undescribed ASA specificities not represented in current commercial panels. Fourth, the semi-quantitative nature of LIA represents an inherent limitation. Although the use of intensity categories aimed to standardize interpretation, inter-assay and inter-run variability may influence BI classification and potentially affect model performance. Finally, the use of final clinical diagnosis as the reference standard may introduce a degree of diagnostic circularity, which is difficult to avoid in complex autoimmune syndromes (29–31). In addition, although the variables included in the model may be biologically related, they were intentionally integrated as complementary diagnostic signals derived from independent laboratory techniques, reflecting real-world clinical interpretation rather than causal inference.

In conclusion, the integration of LIA intensity, compatible cytoplasmic IIF-HEp-2 patterns, and anti-Ro52 co-positivity provides a practical probability-based approach for interpreting ASA-positive LIA results in routine laboratory practice. This strategy may help identify results with higher likelihood of clinical relevance and support decisions regarding further confirmatory testing, particularly when IP is not readily available.

## Data Availability

The raw data supporting the conclusions of this article will be made available by the authors, without undue reservation.
