# Supplementary material for: A multivariable probability score integrating routine immunoassay results for the immunological assessment of antisynthetase syndrome
Source: Front Immunol. 2026 May 29;17:1832694. doi: 10.3389/fimmu.2026.1832694 (PMC13260508; doi:10.3389/fimmu.2026.1832694)
Supplement: Supplementary file 1 [file DataSheet1.docx]

Supplementary Material

# Supplementary Figures and Tables

## Supplementary Tables

Supplementary Table 1. Distribution of Final Diagnoses in the ASA-Positive Control Cohort (n = 92). (i) Interstitial lung diseases (ILD) included idiopathic pulmonary fibrosis, interstitial pneumonia with autoimmune features, hypersensitivity pneumonitis, tobacco-related ILD and combined pulmonary fibrosis–emphysema phenotypes, interstitial lung abnormalities, silicosis, pleuroparenchymal fibroelastosis, diffuse idiopathic pulmonary neuroendocrine cell hyperplasia, and post-COVID organizing pneumonia, as well as cases of undetermined interstitial lung disease not suggestive of antisynthetase syndrome. (ii) Non-ASyS myopathies and neuromuscular disorders comprised immune-mediated necrotizing myopathy, dermatomyositis (including paraneoplastic forms), mitochondrial myopathy, disuse myopathy, isolated hypercreatinkinasemia, myalgia without myositis, proximal myopathy with associated polyneuropathy, myasthenia gravis, and amyotrophic lateral sclerosis. (iii) Systemic autoimmune diseases included Sjögren’s syndrome, systemic sclerosis, rheumatoid arthritis, systemic lupus erythematosus, overlap connective tissue disease, giant cell arteritis, ankylosing spondylitis, Raynaud’s phenomenon, and undifferentiated connective tissue disease. (iv) Non-interstitial pulmonary diseases included chronic obstructive pulmonary disease and bronchiectasis. (v) Neoplasms comprised oropharyngeal and hypopharyngeal squamous cell carcinoma and melanoma associated with checkpoint inhibitor–related myositis. (vi) Infectious diseases included nosocomial and eosinophilic pneumonia. (vii) The miscellaneous group included heterogeneous conditions such as radiculopathy, arthralgia, chronic fatigue syndrome, acute intermittent porphyria, focal postural pathology, and cases with diagnostic uncertainty or under evaluation.

| Diagnostic group | N=92 | % |
| --- | --- | --- |
| Interstitial lung diseases | 33 | 35.9% |
| Myopathies (different from ASYS) / neuromuscular disorders | 16 | 17.4% |
| Systemic autoimmune diseases | 15 | 16.3% |
| Non-interstitial pulmonary diseases | 3 | 3.3% |
| Neoplasms | 4 | 4.3% |
| Infectious diseases | 2 | 2.2% |
| Other / miscellaneous | 19 | 20.7% |

## Supplementary Figures

1. Final logistic regression equation

$$\log\left( \frac{p}{1-p} \right)=\boldsymbol{1.2}\left( {ASA}^{++} \right)+\boldsymbol{3.5}\left( {ASA}^{+++} \right)+\boldsymbol{1.8}\left( IIF-HEp2 AC19/20 \right)+\boldsymbol{2.2}\left( Anti-Ro52 copositivity \right)$$

(B) Definitions of model variables included in the regression equation

| Variable | Definition |
| --- | --- |
| ASA++, ASA+++ | Semi-quantitative ASA reactivity by line-blot immunoassay |
| IIF HEp-2 AC-19/20 | Compatible cytoplasmic pattern by indirect immunofluorescence on HEp-2 cells |
| Anti-Ro52 copositivity | Anti-Ro52 positivity among ASA-positive samples |

**Figure S1. Specification of the multivariable logistic regression model used to derive the laboratory-based diagnostic score.** (A) Final logistic regression equation used to estimate the probability of antisynthetase syndrome (ASS). The model includes semi-quantitative ASA reactivity by line immunoassay (LIA), compatible cytoplasmic pattern on IIF-HEp-2 (AC-19/20), and anti-Ro52 co-positivity. (B) Definitions of model variables included in the regression equation. Reference categories were: ASA-negative (for LIA), negative or non-compatible IIF-HEp-2 pattern, and absence of anti-Ro52 co-positivity. Predicted probabilities derived from this equation were used to generate the ROC curve presented in Figure 3.


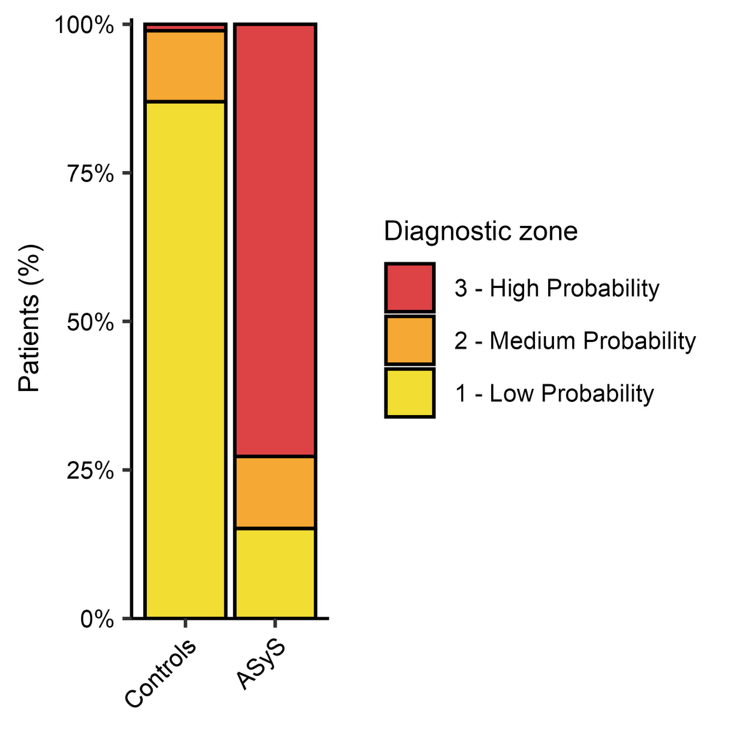


**Figure S2. Distribution of patients across diagnostic probability zones according to final clinical diagnosis.** Stacked bar plot showing the proportion of patients classified into low-, intermediate-, and high-probability zones by the laboratory-based score, stratified by final clinical diagnosis (ASyS vs Controls). The figure illustrates the progressive enrichment of confirmed ASyS cases across increasing probability categories.
